# Topological Properties of Resting-State fMRI Functional Networks Improve Machine Learning-Based Autism Classification

**DOI:** 10.3389/fnins.2018.01018

**Published:** 2019-01-10

**Authors:** Amirali Kazeminejad, Roberto C. Sotero

**Affiliations:** ^1^Hotchkiss Brain Institute, University of Calgary, Calgary, AB, Canada; ^2^Biomedical Engineering Graduate Program, University of Calgary, Calgary, AB, Canada; ^3^Department of Radiology, University of Calgary, Calgary, AB, Canada

**Keywords:** graph theoiy, SVM–support vector machine, machine learing, fMRI, ABIDE, brain connectitvity

## Abstract

Automatic algorithms for disease diagnosis are being thoroughly researched for use in clinical settings. They usually rely on pre-identified biomarkers to highlight the existence of certain problems. However, finding such biomarkers for neurodevelopmental disorders such as Autism Spectrum Disorder (ASD) has challenged researchers for many years. With enough data and computational power, machine learning (ML) algorithms can be used to interpret the data and extract the best biomarkers from thousands of candidates. In this study, we used the fMRI data of 816 individuals enrolled in the Autism Brain Imaging Data Exchange (ABIDE) to introduce a new biomarker extraction pipeline for ASD that relies on the use of graph theoretical metrics of fMRI-based functional connectivity to inform a support vector machine (SVM). Furthermore, we split the dataset into 5 age groups to account for the effect of aging on functional connectivity. Our methodology achieved better results than most state-of-the-art investigations on this dataset with the best model for the >30 years age group achieving an accuracy, sensitivity, and specificity of 95, 97, and 95%, respectively. Our results suggest that measures of centrality provide the highest contribution to the classification power of the models.

## Introduction

Autism Spectrum Disorder (ASD) is a neurodevelopmental disease which manifests in early childhood and persists into adulthood. Recent studies show that 1 in 45 children is diagnosed with autism (Zablotsky et al., 2015). While there is no cure for ASD (Brentani et al., 2013), early diagnosis of autistic individuals is proven to improve quality of life (Fernell et al., 2013). To better detect ASD, biomarkers characterizing the disorder need to be identified. It has been shown that by using topological biomarkers extracted from the brain functional network, machine learning (ML) algorithms can be trained to aid in ASD diagnosis (Plitt et al., 2015). However, there are many variables, such as different methods to construct the functional network and carry out the topological measurements that can affect the extraction of these biomarkers. One goal of this study was to find the best combination of these variables to tackle the task of ASD classification. For this goal, we used 5 different network extraction pipelines with 12 graph theoretical topological measurements and preformed a statistical analysis to compare the classification results between the pipeline. The second goal was to identify the top topological measures in each pipeline and investigate their relation to ASD in order to attempt and further understand the disorder.

Our brains can be viewed as a network of functionally interconnected regions. To measure the strength of these connections, the temporal dynamics of brain activity is needed. Modalities such as Electroencephalography (EEG) and magnetoencephalography (MEG) provide this information, however, they suffer from poor spatial resolution when compared to Functional Magnetic Resonance Imaging (fMRI). In fMRI, brain activity is usually monitored by the changes in blood oxygenation which changes the magnetic properties of blood. The resulting signal is called the Blood-oxygen-level dependent (BOLD) signal. At the turn of the century, researchers provided evidence that fMRI can be used to identify functional connections of the brain while the subject was in a “resting-state” and not doing any specific task (Lowe et al., 2000). Later studies found many different functional networks can be identified using the resting-state connectivity derived from fMRI (van den Heuvel and Hulshoff Pol, 2010). Information from these networks can be extracted and used as an input to ML algorithms to automatically identify the best biomarkers distinguishing between healthy and diseased networks (Nielsen et al., 2013; Plitt et al., 2015; Hazlett et al., 2017; Heinsfeld et al., 2018).

ML has proven to be a powerful tool for automatic disease diagnosis in neurodegenerative disorders such as Alzheimer's Disease (AD) (Chen et al., 2011) and Parkinson's Disease (Kazeminejad et al., 2017; Talai et al., 2017). In recent years, researchers began investigating how the same principles can be used for automatic ASD diagnosis. Promising results with accuracies over 90% were observed using invasive methods and blood analysis (Howsmon et al., 2017). However, the classification studies conducted using non-invasive data acquisition such as brain imaging, while above chance levels, generally report lower accuracies. By using fMRI data acquired in the Autism Brain Imaging Data Exchange (ABIDE) (Nielsen et al., 2013) extracted the pairwise functional connectivity of 7,266 Regions of interest (ROI) using Pearson correlation and used a leave-one-out general linear model classifier to achieve a ASD vs. Healthy Controls (HC) classification accuracy of 60%. More recently, by applying and comparing different ML algorithms to the same dataset, the accuracy has reached 70%. Heinsfeld et al. used the Pearson correlation of fMRI activity of region pairs in CC200 atlas (Craddock et al., 2012) as the inputs to a multi-layer perceptron to achieve this result (Heinsfeld et al., 2018). Other research groups using their own datasets have reported higher accuracies. One study using cortical thickness, total brain volume, and surface area of different brain regions was able to achieve an accuracy of 81% using a neural network as their classifier (Hazlett et al., 2017).

Another emerging methodology in understanding different neurological disorders is graph theory, a mathematical tool used to explain network characteristics that can also be applied to the human brain network (Iturria-Medina et al., 2008; Bullmore and Sporns, 2009; Rubinov and Sporns, 2010; Sotero, 2016; Sanchez-Rodriguez et al., 2018). Graph theory can be used to measure the brain network segregation (clustering coefficient and transitivity), integration (characteristic path length and efficiency), and centrality (betweenness centrality, eigenvector centrality, participation coefficient and within module z-score). Recent brain imaging studies have found topological differences between ASD and normal brains which can be quantified using graph theory, such as global alterations of characteristic path length and efficiency in ASD (Rudie et al., 2013; Itahashi et al., 2014; Zeng et al., 2017; Qin et al., 2018) as well as alterations to segregation measures (Barttfeld et al., 2011; Rudie et al., 2013; Leung et al., 2014; Keown et al., 2017; Zeng et al., 2017) and centrality measures(Di Martino et al., 2013; Leung et al., 2014; Balardin et al., 2015).

Previous studies in AD patients have used topological properties of brain networks as features for a ML algorithm, achieving classification accuracies of 85% (Dyrba et al., 2015). However, this methodology hasn't been tested in ASD. With the emergence of the ABIDE dataset, large amounts of imaging and clinical data has become available to researchers (Di Martino et al., 2014). More than 1,000 datasets are available for individuals with ASD and HC each. This data is collected from multiple sites with slightly varying machinery and imaging parameters. Therefore, a well-developed preprocessing pipeline is essential to minimize the effects of site and imaging parameter changes, but further data manipulations may be needed to standardize the data from different sites.

One explanation for the lower accuracies of studies using the ABIDE dataset is that it covers a large age range (5–65). Age has been proposed as a factor attributing to the different results reported on resting-state fMRI analysis of ASD (Hull et al., 2016). Another study focusing on using multi-scale image textures to study neuroanatomical texture features in autism has found correlations between age and texture features (Chaddad et al., 2017). Therefore, any study that uses all this data will have to take aging effects into consideration. If these issues are correctly addressed, the ABIDE initiative will provide a suitable database for ML centered research on ASD. Another limitation that can be associated with the previously mentioned studies is that they use a simple connectivity matrix such as one computed by Pearson correlation as their features for the classification algorithms. The connectivity matrix is interpreted as the strengths of the connection between ROIs and the changes in these connection strengths are used to classify between ASD and HCs. We hypothesize that by applying graph theoretical measurement of network segregation (clustering coefficient and transitivity), integration (characteristic path length and efficiency), and centrality (betweenness centrality, eigenvector centrality, participation coefficient and within module z-score) for extracting features from the connectivity matrix, the performance of ML algorithm on this dataset will be improved.

In this study, we use fMRI BOLD signals to estimate functional connectivity matrices using different network extraction methods. Using these matrices, we construct a brain network modeling the functional connectivity of a subject's brain. Topological properties such as integration, segregation, and centrality of the obtained networks are then used as features (for a total of 817 features for each network extraction method) fed to a gaussian kernel Support Vector Machine (SVM) to classify whether a subject is suffering from ASD or not. We then use a sequential feature selection technique to choose the top 10 features that contribute to this classification. To control for the effects of aging, we separated our data into 5 age groups. Our best model, for the >30 age range achieved a classification accuracy, sensitivity, and specificity of ~95, 97, and 95%, respectively. Most regions that the features were extracted from had been previously shown to undergo structural and/or functional changes in ASD.

## Materials and Methods

### Dataset and Preprocessing

In order to ensure replicability, we used the preprocessed version of ABIDE I (Di Martino et al., 2014) data publicly available via the Preprocessed Connectome Project (Cameron et al., 2013b). The preprocessing pipeline we used for this study is the Configurable Pipeline for the Analysis of Connectomes (CPAC) (Cameron et al., 2013a). Regions of interests (ROIs) were defined as the 116 regions in the automatic anatomical labeling (AAL) atlas (Tzourio-Mazoyer et al., 2002).

The preprocessing included the following steps. AFNI was used for removing the skull from the images. The brain was segmented into three tissues using FSL. The images were then normalized to the MNI 152 stereotactic space using ANTs. Functional preprocessing included motion and slice-timing correction as well as the normalization of voxel intensity. Nuisance signal regression included 24 parameters for head motion, CompCor with 5 principal components for tissue signal in CSF, and white matter, linear and quadratic trends for Low-frequency drifts and a global bandpass filter (0.01–0.1 Hz). These images where then co-registered to their anatomical counterpart by FSL. They were then normalized to the MNI 152 space using ANTs. The average voxel activity in each ROI was then extracted as the time-series for that region. Any subject that had a consistently 0 time-series was omitted from the dataset. To minimize the effects of age on the results, the dataset was split into 5 age ranges with 5-year increments for the first three step and a 10 year and unlimited increment for the final two. This was done in order to ensure that no age range will have a very small number of subjects. The distribution of the subjects in each age range can be seen in Table 1. Further breakdown of the subject's demographics is shown in Supplementary Table A.

**Table 1:** Distribution of the data.

**Site (# Samples in fMRI time series)**	**5–10**	**10–15**	**15–20**	**20–30**	**30–65**	**All**
	**years**	**years**	**years**	**years**	**years**
CALTECH (146)	0	0	3	13	5	20
CMUA (236)	0	0	0	1	0	1
KKI (152)	20	18	0	0	0	38
LEUVEN1 (246)	0	0	8	21	1	29
LEUVEN2 (246)	0	24	7	0	0	31
MAXMUNA (116)	0	0	1	3	7	11
MAXMUNB (116)	0	0	0	2	5	6
MAXMUNC (116)	0	0	0	13	3	14
MAXMUND (196)	2	5	1	0	2	9
NYU (176)	35	66	26	34	5	166
OHSU (78)	7	15	1	0	0	23
OLIN (206)	0	9	14	7	0	25
PITT (196)	0	16	4	7	5	32
SBL (196)	0	0	0	3	6	8
SDSU (176)	1	17	9	0	0	27
STANFORD (176–236)	21	15	0	0	0	36
TRINITY (146)	0	14	20	9	0	43
UCLA1 (116)	3	38	14	0	0	55
UCLA2 (116)	1	18	1	0	0	20
UM1 (296)	9	42	32	0	0	82
UM2 (296)	0	13	16	2	0	31
USM (236)	0	6	21	22	12	61
YALE (196)	10	26	12	0	0	48
All Sites	109	342	190	137	51	816

*Number of participants from each site for each age group as well as the overall number of participants in a site that were used for this study. Last row shows the total number of subjects in each age-range. The number of MRI samples per fMRI time-series is annotated in brackets in the first column. The Stanford time-series did not have a consistent number of samples thus the number is presented as a range*.

### Creating the Functional Connectivity Network

To extract the whole-brain functional connectivity network of each subject, each ROI is seen as a network node and a measure of connectivity is used to connect these nodes (Bullmore and Sporns, 2009). This connectivity measure (*w*_*ij*_) must be able to quantify the relationship between the time-series of ROI *i* and *j*. Correlation and mutual information metrics have been extensively used for this purpose (Rubinov and Sporns, 2010). We have used spearman's rank correlation coefficient, the percentage-bend correlation (Wilcox, 1994; Pernet et al., 2012) and partial correlation (Marrelec et al., 2006) as our correlation based measures of connectivity. We also used Sparse Inverse Covariance Estimation (SICE) (Huang et al., 2010) and mutual information as alternative measures of connectivity. More details on each method can be found in the Supplementary Material. The implementations used in the open source GraphVar Matlab toolbox (Kruschwitz et al., 2015) was used to compute these connectivity measures.

### Graph Extraction

Once the whole-brain network is available, numerous methods can be used to express it in terms of a graph. The easiest way is to treat each ROI as a node and the connectivity matrix as connection weights. Another approach is to define a threshold *T* and disregard any edges with values *w*_*ij*_<*T* by changing them to 0. One can then either keep the edge weights for *w*_*ij*_>*T* or change them to 1 to construct a binary graph. It has been shown that binary graphs are easier to characterize using graph theoretical metrics and usually have better defined null models for statistical analysis (Rubinov and Sporns, 2010). As there is no proved way to calculate the value of *T* for a specific application, a proportional approach is usually used in its place. In this paper, the highest 20% of the weights were changed to 1 and the rest were disregarded as 0.

### Graph Metrics

Graph theoretical analysis was performed on the extracted brain graph for each subject. The calculated graph properties consisted of measures of segregation (Clustering Coefficient, Transitivity), integration (Characteristic Path Length, Efficiency), and centrality (Betweenness centrality, within module degree Z-score, Participation coefficient) of the brain network. Formulas for each metric are presented in Table A1 (Rubinov and Sporns, 2010). This resulted in a feature space of 817 variables for each subject. More information on this step is available in the Supplementary Material.

All steps from Graph extraction to this point were done using the openly available MATLAB toolbox GraphVar (Kruschwitz et al., 2015).

### Classification, Validation, and Comparison

In this study, we used the python Scikit-learn implementation of the gaussian SVM as our classifier. Features were selected using a sequential forward floating algorithm (Pudil et al., 1994). This was done over 10 successive iterations. In the first iteration, all features in the feature space were individually used for classification and the best performing feature was added to a feature subset while being removed from the feature space. In each consecutive iteration, individual components of the feature space are added to feature subset and the best performing feature in combination with previous results is kept for future use. This resulted in 10 features being chosen as the best graph characteristics that distinguish between ASD and HC.

All classification metrics were acquired using a 10-fold stratified cross validation test with the data folds being the same for all algorithms. To further validate our results, the confusion matrix of each model was evaluated to determine model sensitivity and specificity.

We minimized the risk of overfitting by using three limiting approaches. First, the simplest kernel (linear) was used for the SVM. Second, only 10 features were used to learn to classify between 104 subjects. Finally, using 10-fold cross validation ensured the model is only evaluated on data points that it has not experienced before.

As cross validation is inevitably dependent on how the data was randomly separated, we used a 10 × 10 Welch's *t*-tests to compare our models. The null hypothesis for these tests was that the two models have equal accuracies. To address the issue of multiple comparisons, we also reported the false discovery rate (FDR) corrected *p*-values for these tests.

**Figure 1:**
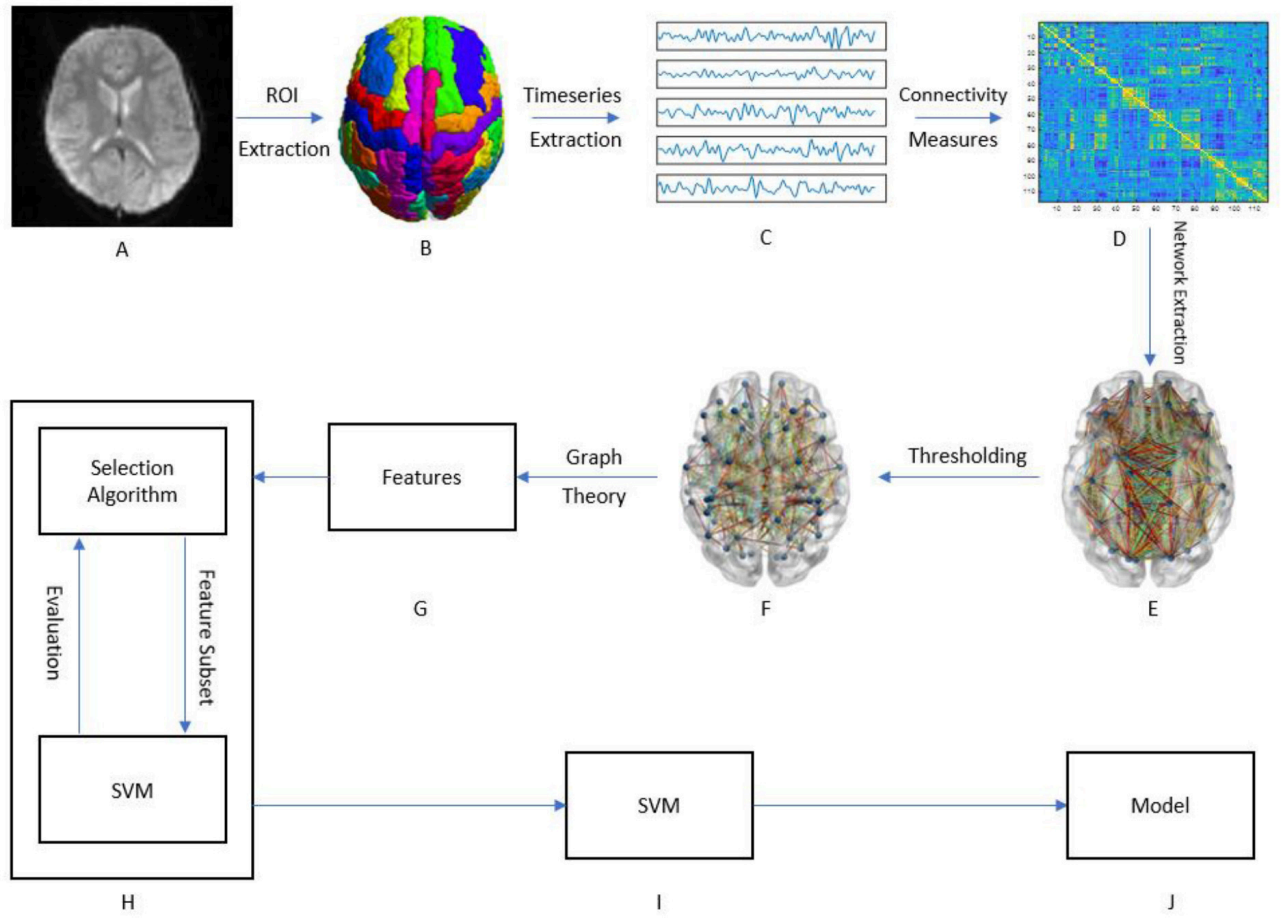
Graphical framework of the experiment. **(A)** Raw fMRI images of subjects; **(B)** After preprocessing the brain is divided into 116 regions of interest (ROI); **(C)** By averaging the BOLD activity in each ROI, a time-series is extracted representing brain activity in that region; **(D)** Using different measures of connectivity, a connectivity matrix is generated from the ROI time-series quantifying the connectivity level between individual ROIs; **(E)** By treating the ROIs as graph nodes and the connectivity matrix as graph weights the brain network is expressed in graph form; **(F)** A threshold is applied to keep only the strongest connections; **(G)** Graph theoretical analysis is applied to the resulting graph from part F to obtain a feature vector for each subject; **(H)** A wrapper method called sequential feature selection is applied to choose a handful of features that contribute to the highest classification accuracy; **(I,J)** The resulting feature subset is passed to a linear SVM which trains a model to distinguish between ASD and HC.

Figure 1 presents a graphical depiction of the methodology proposed here.

## Results

### Performance of the Classifiers

**Figure 2:**
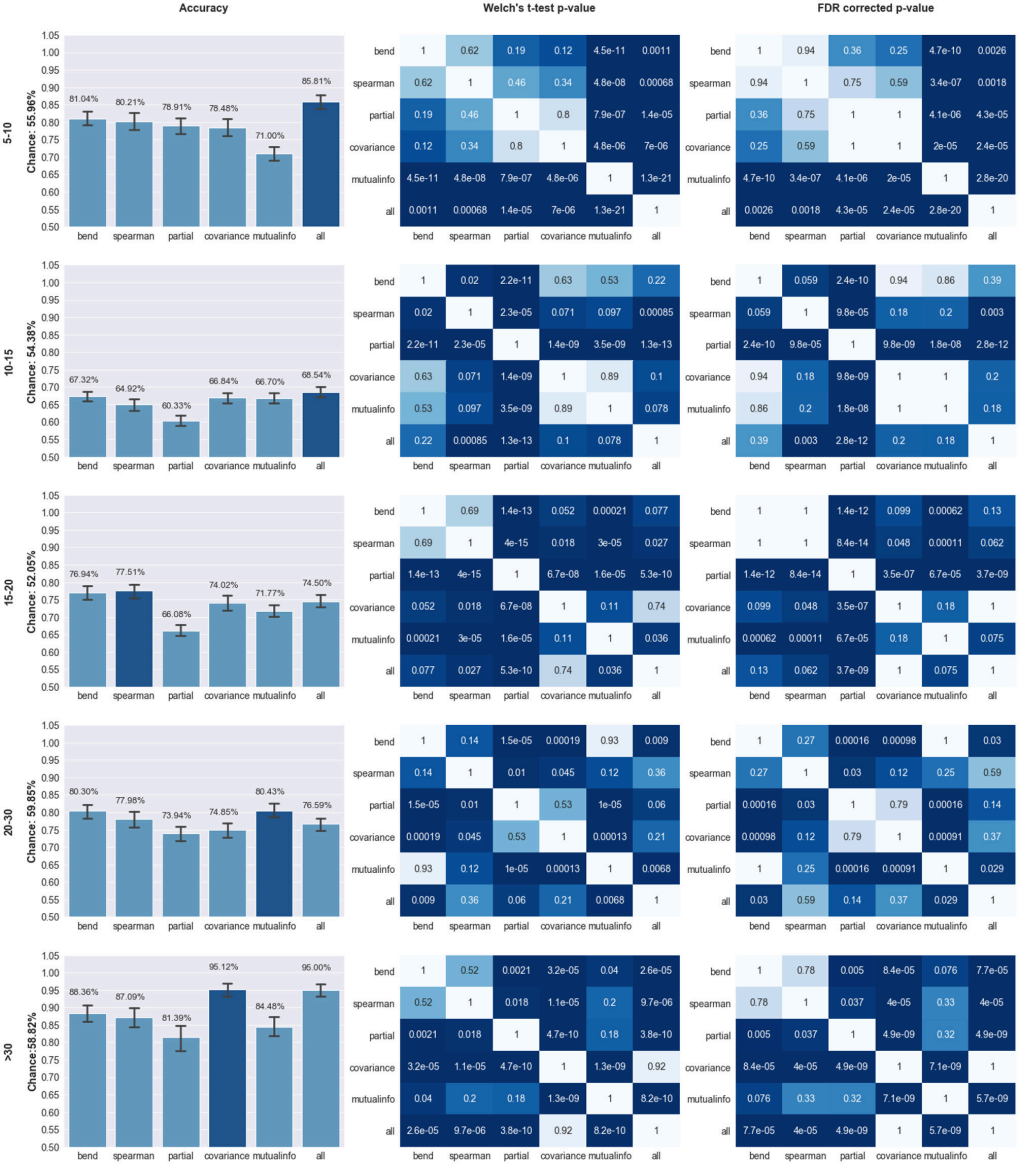
Comparison of Model Performance; Left Column: Accuracy of the models trained using features extracted from the pipeline specified on the X axis for the age range specified on the far left (in years). Y axis labels specify the chance level for the classification task. Top preforming model is highlighted in dark blue; Middle Column: *p*-values of the Welch's *t*-test preformed on the models trained on different pipelines. Statistical significance (*p* < 0.05) is highlighted in dark blue; Right Panel: FDR corrected *p*-values based on the Benjamini, Hochberg method (Benjamini and Hochberg, 1995). The corrected *p*-values were capped at 1 therefore any value over that threshold was set to 1.

Our models were able to consistently perform better than the chance level calculated for their respective age ranges. Chance level was evaluated by assuming the model always chooses the most populous group. The left panel of Figure 2 compares the performance of the different pipelines in each age range. The best preforming model for each age-range is highlighted.

The top preforming pipeline model was generally shown to have a statistically higher (*p* < 0.05) mean than most of the other pipelines. The only exception occurs in the case of the 10–15 age range in which the concatenation pipeline's accuracy fails to achieve a statistically significance difference with three other pipelines: mutual information, covariance, and bend correlation. The details of this statistical analysis are illustrated on the middle and right panels of Figure 2.

To further analyze the performance of the best models, we calculated their respective sensitivity and specificity (Table 2). All models exhibited a specificity of > = 80%. The 10–15 age range showed relatively low sensitivity. Specificity shows the percentage of times that a Negative prediction (in this case HC is correct while sensitivity shows the percentage of times that a Positive prediction (ASD) is correct.

**Table 2:** Classification performance of the best models.

**Age range (years)**	**Best pipeline**	**Accuracy%**	**Specificity%**	**Sensitivity%**
5–10	Concatenation	86	91	79
10–15	Concatenation	69	80	55
15–20	Spearman	78	80	76
20–30	Mutual information	80	87	69
>30	Covariance	95	91	97

### Analysis of Selected Features

**Figure 3:**
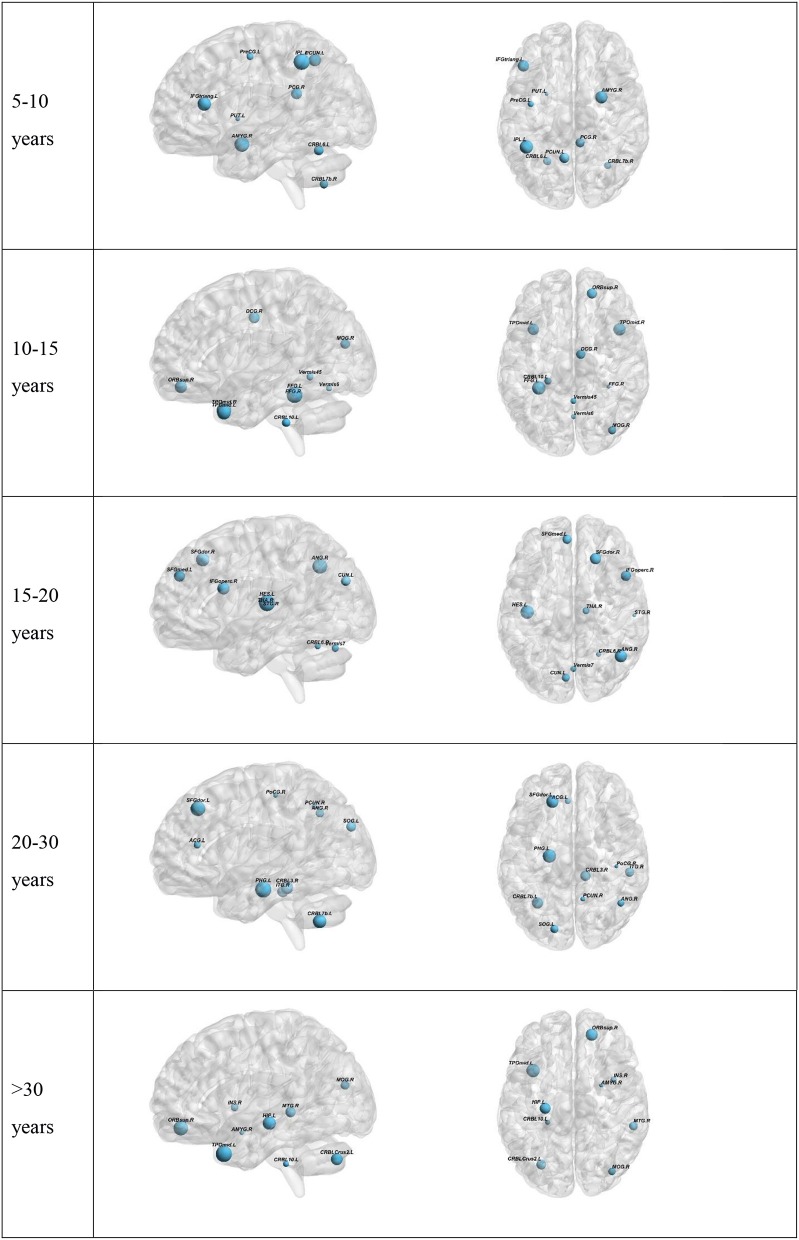
Visualization of the top 10 selected features for each Age range. Two age-ranges show only 9 features. This is because in the 5–10 range PreCG.L was selected two times. In the >30 group the last selected feature was the global Characteristic path length. The full region names along with the abbreviations can be found in Supplementary Table B.

To further understand the results, we plotted the regions from which the selected features were derived (Figure 3). The results for the top-preforming pipeline for each age range will be presented in the main body of this article. More details about the performance of all other pipelines for a given age range is given in the Supplementary Material. The size of the nodes in Figure 3 correspond to the rank at which that feature was selected. The abbreviations of the node labels can be found in the Supplementary Table B. Supplementary Table B also tabulates the exact features for each age range as well as the *p*-value corresponding to the between group difference of that feature. The top group of measures as well as the top measure based on repetition is as follows: Measures of segregation, specifically clustering coefficient for the 5–10 years range. Measures of centrality for all other age ranges, with the most repeated measure being betweenness centrality for the 10–15 years range, eigenvector centrality for the 15–20 years range, within module degree z-score for the 20–30 years range and betweenness centrality for the >30 years range.

## Discussion

### Comparison With Previous Literature

In this study, we examined several different pipelines for ASD classification. These included 6 different network extraction techniques over 5 age ranges. Furthermore, we used 10-fold cross validation to examine the accuracy of the algorithm for each pipeline which is shown to be better than the leave-one-out cross validation used in previous studies (Kohavi, 1995). In addition, 10-fold cross validation may be used as a substitute for having a separate testing set because the model is evaluated on datapoints it has not seen before. Because of not having the exact models trained in previous studies, we compare our findings with them only by using the reported accuracy, specificity, and sensitivity. All models trained in this study were statistically compared with each other using a 10 by 10 cross validation *t*-test.

Previous studies were not able to report high prediction accuracies for the ABIDE dataset relative to similar studies on other neurological diseases such as AD. This can be related to the fact that this dataset consists of recordings conducted over multiple sites, some with slightly different image acquisition parameters. Moreover, the whole dataset covers a wide age range (5–64 years). To minimize the effects of age, we separated the dataset into 5 age ranges and trained separate models on each range. To allow for easier reproducibility and thus more meaningful comparisons, we chose to use a publicly available preprocessed version of the data through the Preprocessed Connectomes Project (http://preprocessed-connectomes-project.org/).

Table 3 shows a detailed comparison with previously reported ASD classification models. It is necessary to state all of the mentioned papers other than Chen et al. (2015) used the complete dataset to train their model while in this study separate models where trained for different age ranges. The cross-validation results in this study provide an estimate of how the models would perform if data from their respective age ranges were fed to them. Therefore, it can be hypothesized that the performance over the entire dataset would not be worse than the worst preforming age-range if, based on the subject's age, the correct model is used for a previously unseen dataset. Additional data is needed to confirm this hypothesis. Our worst preforming model, the model for the 10–15 age range, outperformed almost all the previous models in specificity while having an accuracy comparable to that of the other SVM models. All other age ranges showed higher accuracy than all previous models except the Chen et al. random forest. This could be attributed to the fact that the performance metrics for the random forest model were assessed using a different scheme called out of bag prediction errors as opposed to the cross validation used in our models and all other previously reported studies mentioned here.

**Table 3:** Previous model performance on the ABIDE dataset.

**Accuracy %**	**Sensitivity %**	**Specificity %**	**Algorithm**	**References**
69	72	67	Linear SVM	Plitt et al., 2015
66	60	72	Gaussian SVM	Chen et al., 2015
90.8	89	93	Random forest	Chen et al., 2015
67	NA	NA	SVC	Abraham et al., 2017
70	74	63	Deep neural network	Heinsfeld et al., 2018

### Comparison Between Pipelines

While in all age ranges except the 10–15 range, the top model showed a statistical significance in performance than most of the other models, our results do not reach a consensus about what network creation pipeline preforms best in all cases. However, the bend correlation pipeline's model was the second best model over all age range but the >30 range. Furthermore, it did not show any statistically significant difference in model performance from the top preforming model for the 10–15, 15–20, and 20–30 age ranges. Based on this, we would suggest bend correlation to be the first network construction pipeline for graph theoretical analysis of the ABIDE dataset if computational time is limited.

A possible explanation for the relatively lower performance of the 10–15 range compared to other age ranges is that the larger number of subjects in this group translated into higher between site variability in the data. Therefore, even though our model achieved higher specificity than most previous studies, further steps are needed to address the inherent heterogeneity of the ABIDE dataset.

### Analysis of the Selected Features

Centrality measures were shown to be most operative in providing features for the classification tasks in the top 10 selected features. This also held true when selecting the top 5 features. Centrality measures have been shown to undergo changes in ASD. A previous study on the structural network of the brain found that autism is accompanied by centrality alterations in regions relevant for social and sensorimotor processing (Balardin et al., 2015). Another study found changes in hubness of ASD brain networks using resting-state fMRI (Itahashi et al., 2014). Our results suggest that the changes in centrality measures play a key role in being able to differentiate between ASD and HC. The only exception was observed for the 5–10 years age range where clustering coefficient, a measure of segregation, was chosen more times than the rest. This also held true when only looking at the top 5 features. This suggests that at a young age, there may not be many changes to the hubs of the brain network but the organization of the network into sub-networks is altered.

## Limitations

There are several limitations in the current study. First, ABIDE I data was used in different age ranges to investigate the prediction accuracy of our pipelines while minimizing the effects of aging on the resting-state networks. Furthermore, although to the best of our knowledge ABIDE is the most comprehensive database for ASD functional imaging, further analyses are needed to confirm its representability of the whole ASD population. Second, we relied on a single preprocessing pipeline for the sake of easier comparison between our work and previous studies. It is entirely possible that another preprocessing pipeline is better suited to this graph theoretical approach. Future studies will need to investigate this limitation. Additionally, the comparison between our models and previous studies only used three metrics (accuracy, sensitivity, and specificity). A statistical test may be needed to further analyze the significance of our findings. However, this is not possible without access to the exact cross validation folds or out of bag sample errors of those studies. Nevertheless, due to the observed improvement, we suspect that our algorithm has reached a statistically significant improvement over previous results.

Another shortcoming that is not limited to this study is related to how the classification task is formulated. To the best of our knowledge, all research in this field including the present study have focused on distinguishing HCs from ASDs. However, as the name suggests, ASD is a spectrum and individual cases can vary greatly in how the disorder affects them. To address this issue, databases such as ABIDE will play a vital role. Extensive detailed clinical analysis data will be needed to correctly approximate the position of an individual on the spectrum.

Finally, variability present in the ABIDE dataset, such as different imaging parameters and devices, due to it being a multi-site initiative may lead to uncontrolled variations in the data or model being biased toward better represented sites. While the normalization steps in the preprocessing help reduce the variations, further investigations will be needed to confirm if they have been eliminated to a sufficient degree. Our results show better overall performance over previous investigations which suggests these limitations may have been addressed in a satisfactory manner.

## Conclusion

In this study we utilized graph theory and ML to propose a novel pipeline for automatic diagnosis of ASD which significantly improved performance over previously proposed models. The relative strength of our method suggests graph theoretical analysis paired with the right preprocessing pipeline can nullify the effects of multi-site and multi-device image acquisition to a good degree and is more robust than previous methods. Our pipeline automatically selected 10 biomarkers for each age range being investigated. Measures of centrality were shown to be most operative in distinguishing between ASD and HC.

## Data Availability Statement

The raw data supporting the conclusions of this manuscript will be made available by the authors, without undue reservation, to any qualified researcher.

## Ethics Statement

We used the data collected as part of the ABIDE database and complied with everything that they have asked to be included in any manuscript using that data. The original ethics statement form the (Di Martino et al., 2014) paper is as follows: All contributions were based on studies approved by local IRBs, and data were fully anonymized (removing all 18 HIPAA protected health information identifiers, and face information from structural images). All data distributed were visually inspected prior to release.

## Author Contributions

The work presented here was carried out in collaboration between all authors. The research was designed by both authors. AK acquired and analyzed the data and carried out the experiment with RS providing supervision and guidance. The manuscript was written by AK and revised by RS. All authors have read and approved the submission of the manuscript.

### Conflict of Interest Statement

The authors declare that the research was conducted in the absence of any commercial or financial relationships that could be construed as a potential conflict of interest.
