# Supplementary material for: Topological Properties of Resting-State fMRI Functional Networks Improve Machine Learning-Based Autism Classification
Source: Front Neurosci. 2019 Jan 10;12:1018. doi: 10.3389/fnins.2018.01018 (PMC6335365; doi:10.3389/fnins.2018.01018)
Supplement: Supplementary file 1 [file Data_Sheet_1.docx]

**Use of topological properties of resting-state fMRI functional networks improves machine learning-based autism classification**

**Amirali Kazeminejad^1,2^, and Roberto C. Sotero^1,3^**

^1^Hotchkiss Brain Institute, University of Calgary, Calgary, AB, Canada

^2^Biomedical Engineering Graduate Program, University of Calgary, Calgary, AB, Canada

^3^Department of Radiology, University of Calgary, Calgary, AB, Canada

***Correspondence:**Amirali Kazeminejad
amirali.kazeminejad@ucalgary.ca

**Email:** [amirali.kazeminejad@ucalgary.ca](mailto:amirali.kazeminejad@ucalgary.ca), roberto.soterodiaz@ucalgary.ca

**Graph Metrics**

Measures of integration were as followed. Global Characteristic Path Length $\left( L \right)$is the average shortest path between any two nodes in the network. Shortest path is defined by the least number of edges it takes to travel from one node to another. The same metric can be computed locally $\left( L_{i} \right)$ by averaging the shortest path of a specific node to all other nodes in the network. Global Efficiency $\left( E \right)$ is defined by the average inverse shortest path between any two nodes and can be computed locally$\left( E_{loc} \right)$ using the same definition by treating the neighborhood of each node as a sub-network.

Segregation can be quantified by the following metrics. Local Clustering Coefficient $\left( C_{i} \right)$ is calculated by taking the fraction of a node’s neighbors (nodes that are connected by edges to the examined node) that are neighbors of each other to all neighbors. It can also be defined by the number of triangles that a node forms with its neighbors divided by the total number of possible triangles between them. To calculate it globally, the normalized Clustering Coefficient $\left( C \right)$ of all the nodes are used. Thus, high global clustering coefficient represents a network consisting of many modules. The formulation for global clustering coefficients leads to each node being normalized by its own nodal degree. This will mean nodes with low nodal degree will have a higher contribution to the overall clustering coefficient. Transitivity $\left( T \right)$ is a measure of segregation designed to minimize the influence of nodal degree by normalizing all individual clustering coefficients at the same time.

One of the metrics that captures the topology of a network is Small-Worldness $\left( S \right)$. Small-world networks are characterized by low characteristic path lengths and high clustering coefficients hinting at high modularity and efficiency. To quantify Small-Worldness, the ratio of the Clustering Coefficient of the network to that of a random network with the same number of nodes is divided by the ratio of the Characteristic Path Length of the network to that of the same random network. In this study, a metric called Small World Propensity was calculated which quantifies small-wordless without dependency on graph density (Muldoon, Bridgeford, and Bassett 2016).

Finally, several centrality measures were calculated. Betweenness Centrality$\left( BC\left( v \right) \right)$ is local metric counting the number of shortest paths of the network that go through a node $v$. Within Module Z-score $z_{i}$ is another local measure of centrality. It quantifies the centrality of each node within its own subnetwork or module. Participation Coefficient $y_{i}$ quantifies the centrality of a node in a module to that of the nodes in another module. Thus, a node with high within module Z-score is an important node for within module information flow while a high participation coefficient signifies importance in between module communications. Finally, Eigenvector $x_{v}$ centrality is another measure of a nodes “hubness” which was studied in this investigation.

**Connectivity measures**

Pearson’s Product Moment Correlation is one of the most widely used measure of functional connectivity in medical research (Mukaka 2012). It attempts to fit a line through the data of two variables and the coefficient *r* denotes how well this line fits the data by measuring how far the data points are relative to the line. This leaves Pearson correlation to be susceptible to extreme values.

Spearman’s Rank Correlation Coefficient measures whether the two variables have a monotonic relationship or not. If the value of one variable increases (decreases) and at the same time an increase (decrease) of the other variable’s value is observed, their relationship is considered monotonic. Thus, Spearman correlation is less influenced by extreme values and can perform correctly in case of their presence.

The Percentage-Bend Correlation is another proposed method to alleviate the shortcoming of Pearson’s Correlation on datasets with extreme marginal values (Wilcox 1994). First, the top *x* percentage of values deviating from the median are down-weighted. This ensures that there are fewer extreme marginal values present in the data. Then a normal Pearson correlation is calculated for the altered data. For this research, we chose the percentage to be 20% in accordance to a previous study (Pernet, Wilcox, and Rousselet 2012).

A shortcoming of all previous methods is that they only consider one pair of brain regions at any given time. Each of these regions can be affected by other brain regions at any given time which can lead to a higher or lower correlation score than if the two regions were isolated. Partial Correlation addresses this problem by considering the effect of other ROIs on the examined regions. A simple way to calculate Partial Correlation is by eliminating any outside effects using linear regression. However, this will be very computationally expensive as the task will have to be repeated for each pair of variables. In practice, it is calculated by using the inverse covariance matrix (Marrelec et al. 2006).

Sparse Inverse Covariance Estimation (SICE) is a direct progression from partial correlation that expands its usefulness to smaller datasets (Huang et al. 2010).

Finally, Mutual Information is an information theory metric that quantifies how much information we can learn about a variable using another variable.

We used the GraphVar toolbox implementations to calculate each of the mentioned connectivity measures (Kruschwitz et al. 2015)

Supplementary Table A. Showing the age mean-standard deviation as well as the number of subjects for each diagnostic group in each site for each age-range. SD: Standard Deviation, nan means that it could not be computed

| **Age range** | **Site** | **Group** | **AGE mean\|sd** | **Count** |
| --- | --- | --- | --- | --- |
| **5, 10** | **KKI** | **ASD** | 8.29 \| 0.16 | 6 |
|  |  | **HC** | 9.34 \| 0.53 | 14 |
|  | **MAXMUND** | **HC** | 8.50 \| 2.12 | 2 |
|  | **NYU** | **ASD** | 8.68 \| 1.00 | 19 |
|  |  | **HC** | 8.32 \| 0.89 | 16 |
|  | **OHSU** | **ASD** | 9.22 \| 0.82 | 4 |
|  |  | **HC** | 9.01 \| 0.75 | 3 |
|  | **SDSU** | **HC** | 8.67 \| nan | 1 |
|  | **STANFORD** | **ASD** | 8.78 \| 0.78 | 9 |
|  |  | **HC** | 8.81 \| 0.61 | 12 |
|  | **UCLA1** | **ASD** | 8.88 \| 0.54 | 2 |
|  |  | **HC** | 9.50 \| nan | 1 |
|  | **UCLA2** | **HC** | 9.79 \| nan | 1 |
|  | **UM1** | **ASD** | 9.57 \| 0.25 | 4 |
|  |  | **HC** | 9.40 \| 0.68 | 5 |
|  | **YALE** | **ASD** | 8.17 \| 1.23 | 4 |
|  |  | **HC** | 8.90 \| 0.81 | 6 |
| **10, 15** | **KKI** | **ASD** | 10.82 \| 0.69 | 6 |
|  |  | **HC** | 11.05 \| 0.85 | 12 |
|  | **LEUVEN2** | **ASD** | 13.68 \| 1.01 | 12 |
|  |  | **HC** | 13.39 \| 0.91 | 12 |
|  | **MAXMUND** | **ASD** | 11.00 \| 0.00 | 3 |
|  |  | **HC** | 11.00 \| nan | 1 |
|  | **NYU** | **ASD** | 12.39 \| 1.54 | 29 |
|  |  | **HC** | 12.45 \| 1.38 | 37 |
|  | **OHSU** | **ASD** | 12.15 \| 1.45 | 7 |
|  |  | **HC** | 10.88 \| 0.69 | 8 |
|  | **OLIN** | **ASD** | 12.80 \| 1.64 | 5 |
|  |  | **HC** | 14.50 \| 0.58 | 4 |
|  | **PITT** | **ASD** | 13.11 \| 0.95 | 9 |
|  |  | **HC** | 13.10 \| 1.02 | 7 |
|  | **SDSU** | **ASD** | 13.34 \| 0.90 | 5 |
|  |  | **HC** | 13.87 \| 0.86 | 12 |
|  | **STANFORD** | **ASD** | 11.70 \| 0.60 | 8 |
|  |  | **HC** | 11.74 \| 0.91 | 7 |
|  | **TRINITY** | **ASD** | 13.58 \| 0.82 | 6 |
|  |  | **HC** | 13.82 \| 1.09 | 8 |
|  | **UCLA1** | **ASD** | 12.56 \| 1.61 | 17 |
|  |  | **HC** | 13.02 \| 1.19 | 21 |
|  | **UCLA2** | **ASD** | 11.76 \| 1.32 | 7 |
|  |  | **HC** | 12.63 \| 0.64 | 11 |
|  | **UM1** | **ASD** | 12.78 \| 1.25 | 22 |
|  |  | **HC** | 12.29 \| 1.46 | 20 |
|  | **UM2** | **ASD** | 13.82 \| 0.80 | 6 |
|  |  | **HC** | 14.13 \| 0.62 | 7 |
|  | **USM** | **ASD** | 11.84 \| 0.69 | 2 |
|  |  | **HC** | 13.04 \| 1.90 | 4 |
|  | **YALE** | **ASD** | 12.76 \| 1.25 | 12 |
|  |  | **HC** | 12.87 \| 1.45 | 14 |
| **15, 20** | **CALTECH** | **ASD** | 17.50 \| nan | 1 |
|  |  | **HC** | 19.35 \| 0.92 | 2 |
|  | **LEUVEN1** | **ASD** | 19.00 \| 0.58 | 7 |
|  |  | **HC** | 18.00 \| nan | 1 |
|  | **LEUVEN2** | **ASD** | 15.30 \| nan | 1 |
|  |  | **HC** | 15.93 \| 0.75 | 6 |
|  | **MAXMUNA** | **ASD** | 19.00 \| nan | 1 |
|  | **MAXMUND** | **ASD** | 18.00 \| nan | 1 |
|  | **NYU** | **ASD** | 16.96 \| 1.58 | 8 |
|  |  | **HC** | 16.97 \| 1.41 | 18 |
|  | **OHSU** | **ASD** | 15.23 \| nan | 1 |
|  | **OLIN** | **ASD** | 18.00 \| 1.53 | 7 |
|  |  | **HC** | 17.50 \| 1.73 | 4 |
|  | **PITT** | **ASD** | 16.99 \| 0.00 | 2 |
|  |  | **HC** | 16.24 \| 1.26 | 2 |
|  | **SDSU** | **ASD** | 16.14 \| 0.55 | 4 |
|  |  | **HC** | 15.97 \| 0.62 | 5 |
|  | **TRINITY** | **ASD** | 17.17 \| 1.70 | 11 |
|  |  | **HC** | 17.72 \| 1.68 | 9 |
|  | **UCLA1** | **ASD** | 16.66 \| 0.93 | 9 |
|  |  | **HC** | 16.45 \| 1.26 | 5 |
|  | **UCLA2** | **ASD** | 16.47 \| nan | 1 |
|  | **UM1** | **ASD** | 16.45 \| 1.12 | 10 |
|  |  | **HC** | 17.26 \| 1.20 | 21 |
|  | **UM2** | **ASD** | 16.28 \| 0.76 | 6 |
|  |  | **HC** | 16.74 \| 1.04 | 10 |
|  | **USM** | **ASD** | 17.69 \| 1.01 | 13 |
|  |  | **HC** | 17.66 \| 1.52 | 8 |
|  | **YALE** | **ASD** | 16.74 \| 0.62 | 6 |
|  |  | **HC** | 16.34 \| 0.91 | 6 |
| **20, 30** | **CALTECH** | **ASD** | 22.12 \| 3.91 | 5 |
|  |  | **HC** | 23.99 \| 3.31 | 7 |
|  | **CMUA** | **ASD** | 22.00 \| nan | 1 |
|  | **LEUVEN1** | **ASD** | 23.50 \| 2.88 | 6 |
|  |  | **HC** | 23.64 \| 2.62 | 14 |
|  | **MAXMUNA** | **ASD** | 27.50 \| 2.12 | 2 |
|  |  | **HC** | 23.00 \| nan | 1 |
|  | **MAXMUNB** | **ASD** | 29.50 \| 0.71 | 2 |
|  | **MAXMUNC** | **ASD** | 22.00 \| nan | 1 |
|  |  | **HC** | 25.75 \| 3.05 | 12 |
|  | **NYU** | **ASD** | 24.06 \| 2.97 | 13 |
|  |  | **HC** | 23.46 \| 2.77 | 21 |
|  | **OLIN** | **ASD** | 22.50 \| 2.12 | 2 |
|  |  | **HC** | 21.67 \| 1.15 | 3 |
|  | **PITT** | **ASD** | 21.16 \| 0.46 | 2 |
|  |  | **HC** | 22.94 \| 1.98 | 5 |
|  | **SBL** | **ASD** | 30.00 \| nan | 1 |
|  |  | **HC** | 26.50 \| 0.71 | 2 |
|  | **TRINITY** | **ASD** | 21.71 \| 0.98 | 4 |
|  |  | **HC** | 22.45 \| 2.61 | 5 |
|  | **UM2** | **HC** | 27.80 \| 1.41 | 2 |
|  | **USM** | **ASD** | 24.93 \| 3.00 | 14 |
|  |  | **HC** | 26.28 \| 2.18 | 8 |
| **30, 65** | **CALTECH** | **ASD** | 46.55 \| 12.52 | 2 |
|  |  | **HC** | 46.00 \| 8.98 | 3 |
|  | **LEUVEN1** | **ASD** | 32.00 \| nan | 1 |
|  | **MAXMUNA** | **ASD** | 39.00 \| 8.49 | 2 |
|  |  | **HC** | 35.00 \| 6.16 | 5 |
|  | **MAXMUNB** | **ASD** | 46.00 \| 11.43 | 4 |
|  | **MAXMUNC** | **HC** | 35.00 \| nan | 1 |
|  | **MAXMUND** | **ASD** | 40.00 \| 7.07 | 2 |
|  | **NYU** | **ASD** | 38.93 \| 0.24 | 2 |
|  |  | **HC** | 30.88 \| 0.85 | 3 |
|  | **PITT** | **ASD** | 33.31 \| 1.54 | 4 |
|  |  | **HC** | 33.17 \| nan | 1 |
|  | **SBL** | **ASD** | 47.50 \| 23.33 | 2 |
|  |  | **HC** | 38.67 \| 3.06 | 3 |
|  | **USM** | **ASD** | 36.89 \| 5.91 | 9 |
|  |  | **HC** | 36.64 \| 4.64 | 3 |

Supplementary Table B1. Selected features for age range 5-10 years and previous studies reporting structural or functional changes for that region

| **Pipeline** | **Graph Metric** | **Region** | **Abbreviation** | **p-value** |
| --- | --- | --- | --- | --- |
| Spearman | Clustering Coefficient | Inferior parietal, but supramarginal and angular gyri | IPL.L | 0.0368089 |
| Covariance | Within-module degree z-score | Amygdala (Right) | AMYG.R | 0.028559 |
| Spearman | Clustering Coefficient | Inferior frontal gyrus, triangular part (Left) | IFGtriang.L | 0.0159884 |
| Mutual information | Characteristic path Length | Precuneus (Left) | PCUN.L | 0.000668882 |
| Covariance | Clustering Coefficient | Posterior cingulate gyrus (Right) | PCG.R | 0.025256 |
| Spearman | Efficiency | Cerebelum_6_L | CRBL6.L | 0.243076 |
| Covariance | Betweenness Centrality | Cerebelum_7b_R | CRBL7b.R | 0.906727 |
| Covariance | Clustering Coefficient | Precentral gyrus (Left) | PreCG.L | 0.355829 |
| Mutual information | Efficiency | Precentral gyrus (Left) | PreCG.L | 0.0209225 |
| Partial | Within-module degree z-score | Lenticular nucleus, putamen (Left) | PUT.L | 0.118273 |

Supplementary Table B2. Selected features for age range 10-15 years and previous studies reporting structural or functional changes for that region

| **Pipeline** | **Graph Metric** | **Region** | **Abbreviation** | **p-value** |
| --- | --- | --- | --- | --- |
| Covariance | Betweenness Centrality | Fusiform gyrus (Left) | FFG.L | 0.0257671 |
| Covariance | Betweenness Centrality | Temporal pole: middle temporal gyrus (Right) | TPOmid.R | 0.00416598 |
| Mutual information | Efficiency | Temporal pole: middle temporal gyrus (Left) | TPOmid.L | 0.912742 |
| Bend | Betweenness Centrality | Superior frontal gyrus, orbital part (Right) | ORBsup.R | 0.542448 |
| Partial | Betweenness Centrality | Median cingulate and paracingulate gyri (Right) | DCG.R | 0.143617 |
| Covariance | Within-module degree z-score | Middle occipital gyrus (Right) | MOG.R | 0.0776933 |
| Partial | Characteristic path Length | Cerebelum_10_L | CRBL10.L | 0.387885 |
| Mutual information | Clustering Coefficient | Vermis_4_5 | Vermis45 | 0.916132 |
| Partial | Within-module degree z-score | Vermis_6 | Vermis6 | 0.533312 |
| Covariance | Participation Coefficient | Fusiform gyrus (Right) | FFG.R | 0.260798 |

Supplementary Table B3. Selected features for age range 15-20 years and previous studies reporting structural or functional changes for that region

| **Pipeline** | **Graph Metric** | **Region** | **Abbreviation** | **p-value** |
| --- | --- | --- | --- | --- |
| Spearman | Characteristic path Length | Heschl gyrus (Left) | HES.L | 7.15E-05 |
| Spearman | Clustering Coefficient | Angular gyrus (Right) | ANG.R | 0.000167317 |
| Spearman | Eigenvector Centrality | Superior frontal gyrus, dorsolateral (Right) | SFGdor.R | 0.273987 |
| Spearman | Eigenvector Centrality | Inferior frontal gyrus, opercular part (Right) | IFGoperc.R | 0.486142 |
| Spearman | Eigenvector Centrality | Superior frontal gyrus, medial (Left) | SFGmed.L | 0.423809 |
| Spearman | Eigenvector Centrality | Cuneus (Left) | CUN.L | 0.145407 |
| Spearman | Within-module degree z-score | Thalamus (Right) | THA.R | 0.0300153 |
| Spearman | Clustering Coefficient | Vermis_7 | Vermis7 | 0.607888 |
| Spearman | Within-module degree z-score | Cerebelum_6_R | CRBL6.R | 0.00123175 |
| Spearman | Characteristic path Length | Superior temporal gyrus (Right) | STG.R | 0.256614 |

Supplementary Table B4. Selected features for age range 20-30 years and previous studies reporting structural or functional changes for that region

| **Pipeline** | **Graph Metric** | **Region** | **Abbreviation** | **p-value** |
| --- | --- | --- | --- | --- |
| Mutual Information | Within-module degree z-score | Parahippocampal gyrus (Left) | PHG.L | 0.154213 |
| Mutual Information | Within-module degree z-score | Superior frontal gyrus, dorsolateral (Left) | SFGdor.L | 0.0145346 |
| Mutual Information | Participation Coefficient | Cerebelum_7b_L | CRBL7b.L | 0.00953836 |
| Mutual Information | Betweenness Centrality | Cerebelum_3_R | CRBL3.R | 0.172049 |
| Mutual Information | Characteristic path Length | Inferior temporal gyrus (Right) | ITG.R | 0.153338 |
| Mutual Information | Betweenness Centrality | Superior occipital gyrus (Left) | SOG.L | 0.767554 |
| Mutual Information | Efficiency | Angular gyrus (Right) | ANG.R | 0.499045 |
| Mutual Information | Efficiency | Anterior cingulate and paracingulate gyri (Left) | ACG.L | 0.394514 |
| Mutual Information | Within-module degree z-score | Precuneus (Right) | PCUN.R | 0.958967 |
| Mutual Information | Within-module degree z-score | Postcentral gyrus (Right) | PoCG.R | 0.761263 |

Supplementary Table B5. Selected features for age range >30 years and previous studies reporting structural or functional changes for that region

| **Pipeline** | **Graph Metric** | **Region** | **Abbreviation** | **p-value** |
| --- | --- | --- | --- | --- |
| Covariance | Characteristic path Length | Temporal pole: middle temporal gyrus (Left) | TPOmid.L | 0.00124389 |
| Covariance | Eigenvector Centrality | Superior frontal gyrus, orbital part (Right) | ORBsup.R | 0.871748 |
| Covariance | Betweenness Centrality | Hippocampus (Left) | HIP.L | 0.0330782 |
| Covariance | participation_coefficient2 | Cerebelum_Crus2_L | CRBLCrus2.L | 0.504822 |
| Covariance | Within-module degree z-score | Middle temporal gyrus (Right) | MTG.R | 0.146737 |
| Covariance | Betweenness Centrality | Middle occipital gyrus (Right) | MOG.R | 0.20807 |
| Covariance | Eigenvector Centrality | Insula (Right) | INS.R | 0.922012 |
| Covariance | Efficiency | Cerebelum_10_L | CRBL10.L | 0.261675 |
| Covariance | Betweenness Centrality | Amygdala (Right) | AMYG.R | 0.135061 |
| Covariance | Global Characteristic Path Length |  |  | 0.474429 |

Huang, Shuai, Jing Li, Liang Sun, Jieping Ye, Adam Fleisher, Teresa Wu, Kewei Chen, and Eric Reiman. 2010. “Learning Brain Connectivity of Alzheimer’s Disease by Sparse Inverse Covariance Estimation.” *NeuroImage* 50 (3): 935–49. https://doi.org/10.1016/j.neuroimage.2009.12.120.

Kruschwitz, J D, D List, L Waller, M Rubinov, and H Walter. 2015. “GraphVar: A User-Friendly Toolbox for Comprehensive Graph Analyses of Functional Brain Connectivity.” *Journal of Neuroscience Methods* 245: 107–15. https://doi.org/10.1016/j.jneumeth.2015.02.021.

Marrelec, Guillaume, Alexandre Krainik, Hugues Duffau, Mélanie Pélégrini-Issac, Stéphane Lehéricy, Julien Doyon, and Habib Benali. 2006. “Partial Correlation for Functional Brain Interactivity Investigation in Functional MRI.” https://doi.org/10.1016/j.neuroimage.2005.12.057.

Mukaka, M M. 2012. “Statistics Corner: A Guide to Appropriate Use of Correlation Coefficient in Medical Research Definitions of Correlation and Clarifications.” *Malawi Medical Journal* 24 (3): 69–71. https://www.ncbi.nlm.nih.gov/pmc/articles/PMC3576830/pdf/MMJ2403-0069.pdf.

Muldoon, Sarah Feldt, Eric W. Bridgeford, and Danielle S. Bassett. 2016. “Small-World Propensity and Weighted Brain Networks.” *Scientific Reports* 6 (1): 22057. https://doi.org/10.1038/srep22057.

Pernet, Cyril R, Rand Wilcox, and Guillaume A Rousselet. 2012. “Robust Correlation Analyses: False Positive and Power Validation Using a New Open Source Matlab Toolbox.” *Frontiers in Psychology* 3. Frontiers Media SA: 606. https://doi.org/10.3389/fpsyg.2012.00606.

Wilcox, Rand R. 1994. “The Percentage Bend Correlation Coefficient.” *Psychometrika* 59 (4). Springer-Verlag: 601–16. https://doi.org/10.1007/BF02294395.
