# Supplementary material for: Topological Properties of Resting-State fMRI Functional Networks Improve Machine Learning-Based Autism Classification
Source: Front Neurosci. 2019 Jan 10;12:1018. doi: 10.3389/fnins.2018.01018 (PMC6335365; doi:10.3389/fnins.2018.01018)
Supplement: Supplementary file 2 [file Data_Sheet_2.docx]

**Table A.1**. Graph theoretical metrics used in this study and their formulae

| **Graph Metric** | **Description** | **Formula** |
| --- | --- | --- |
| Betweenness Centrality | The fraction of shortest paths in network that contain a given node | $BC\left( \nu\right)=\sum_{\begin{matrix} u,w\in N \\ u\neq w\neq\upsilon\end{matrix}} \frac{\sigma_{uw}\left( \nu\right)}{\sigma_{uw}}$  $\sigma_{uw}$: Number of shortest paths between u and w  $\sigma_{uw}\left( \nu\right)$: Number of shortest path between u and w that pass through v |
| Characteristic Path Length Global | Average shortest path between any two nodes in the network | $L=\frac{1}{n}\sum_{i\in N} L_{i}$  $L_{i}$: Local characteristic path length |
| Characteristic Path Length Local | Average of the shortest path of an individual node to all other nodes | $L_{i}=\frac{\sum_{j\in N} L_{ij}}{n-1}$  $L_{ij}$: Shortest path length (number of edges) between nodes i and j |
| Clustering Coefficient Global | Average of the local clustering coefficient of all nodes | $C=\frac{1}{n}\sum_{i\in N} C_{i}$ |
| Clustering Coefficient Local | fractions of a node’s neighbors (connected) that are neighbors of each other | $C_{i}=\frac{2t_{i}}{k_{i}\left( k_{i}-1 \right)}$  $t_{i}=\frac{1}{2}\sum_{j,h\in N} a_{ij}a_{ih}a_{jh}$  $t_{i}$: Number of triangles around node i  $a_{ij}$: Is one if there is a connection from node i to j and is zero otherwise |
| Efficiency Global | Average inverse shortest path between any two nodes | $E=\frac{1}{n}\sum_{i\in N} E_{i}$  $E_{i}=\frac{\sum_{j\in N,j\neq i} L_{ij}^{-1}}{n-1}$ |
| Efficiency Local | Global efficiency calculated in the neighborhood each node | $E_{loc}=\frac{1}{n}\sum_{i\in N} E_{loc,i}$  $\frac{\sum_{j,h\in N,j\neq i} a_{ij}a_{ih}\left[ L_{jh}\left( N_{i} \right) \right]^{-1}}{k_{i}\left( k_{i}-1 \right)}$  $L_{jh}\left( N_{i} \right)$: Shortest path between j and h in the neighborhood of node i |
| Eigenvector Centrality | Self-referential measure of centrality | $x_{v}=\frac{1}{\lambda}\sum_{j\in M_{v}} a_{ij}x_{j}$  $M_{v}$**:** Neighborhood of node v |
| Module Degree Z-score | Within module measure of centrality (evaluated in individual neighborhoods/modules) | $z_{i}=\frac{k_{i}\left( m_{i} \right)-\left( k\left( m_{i} \right) \right)}{\sigma^{k\left( z_{i} \right)}}$  $m_{i}$: Module containing node i  $k_{i}\left( m_{i} \right)$: Within module degree of node i  $k\left( m_{i} \right)\wedge\sigma^{k\left( z_{i} \right)}$: Mean and SD of within $m_{i}$ degree distribution |
| Participation Coefficient | Measure of diversity of intermodular connections | $y_{i}=1-\sum_{m\in M} \left( \frac{k_{i}\left( m \right)}{k_{i}} \right)^{2}$  $M:$ Set of all modules |
| Small World Propensity | Measure of small-worldness of the network | $S=\frac{\frac{C}{C_{rand}}}{\frac{L}{L_{rand}}}$  $C_{rand}\wedge L_{rand}$: C and L for a random network with the same number of nodes |
| Transitivity | Fraction of triangles to triplets in the global network | $T=\frac{\sum_{i\in N} 2t_{i}}{\sum_{i\in N} k_{i}\left( k_{i}-1 \right)}$  $k_{i}$: Node degree |
